# Proximal anatomical axes show bidirectional variability: Accurate tibial slope requires mechanical axis measurement

**DOI:** 10.1002/ksa.70093

**Published:** 2025-10-22

**Authors:** Barbara C. Boer, Quinten W. T. Veerman, Reinoud W. Brouwer, Judith olde Heuvel, Tony G. van Tienen, Wolf Petersen, Roy A. G. Hoogeslag

**Affiliations:** ^1^ OCON Centre for Orthopaedic Surgery and Sports Medicine Hengelo the Netherlands; ^2^ Department of Biomechanical Engineering, Faculty of Engineering Technology University of Twente Enschede the Netherlands; ^3^ Martini Ziekenhuis Groningen the Netherlands; ^4^ Kliniek Viasana Mill the Netherlands; ^5^ Klinik für Orthopädie und Unfallchirurgie Martin Luther Krankenhaus Berlin Germany

**Keywords:** anatomical axis, mechanical axis, posterior tibial slope, radiologic measurement, tibial axis

## Abstract

**Purpose:**

To determine whether posterior tibial slope (PTS) measured to proximal anatomical axes can serve as a reliable proxy for PTS measured to the mechanical tibial axis (mTA).

**Methods:**

In patients ≥18 years with failed anterior cruciate ligament reconstruction, medial and lateral PTS were measured relative to the mTA on a lateral lower‐leg radiograph, and relative to the proximal tibial anatomical axis (PTAA), anterior tibial cortical axis (ATC) and posterior tibial cortical axis (PTC) on a lateral knee radiograph. Agreement was assessed using correlation coefficients, measurement error and Bland–Altman analysis. Diagnostic accuracy of the conventional ≥12° PTAA‐based PTS cut‐off for slope‐reducing osteotomy was determined.

**Results:**

Fifty‐nine knees were analyzed. Correlation between proximal anatomical axes and mTA‐based PTS measurements was strong (*r* ≥ 0.71), but agreement was poor. Mean measurement error for PTAA was 0.1 ± 2.4° (range: −5.7° to 7.6°) and −0.5 ± 2.4° (range: −6.6° to 5.2°), for ATC −3.4 ± 2.6° (range: −8.6° to 5.9°) and −3.7 ± 2.6° (range: −9.9° to 2.7°) and for PTC 3.0 ± 2.6° (range: −2.9° to 10.4°) and 2.7 ± 2.4° (range: −3.6° to 7.8°) for the medial and lateral plateau, respectively. For PTAA, absolute measurement error ≥2° occurred in approximately 50% of knees. Bland–Altman analysis showed 95% levels of agreement ranging from −4.89° to 4.62° (medial) and −5.11° to 4.11° (lateral). Using the ≥12° cut‐off on PTAA‐based PTS values misclassified approximately one in three knees (19.5% false‐positive and 13.2% false‐negative) relative to the mTA‐based PTS reference values.

**Conclusion:**

Despite strong correlation, proximal anatomical axes‐based PTS measurements on routine lateral knee radiographs show substantial, bidirectional measurement error outside clinically acceptable limits of agreement compared to mTA‐based PTS measurements on full‐length lateral lower‐leg radiographs. Single‐exposure, full‐length lateral lower‐leg radiographs should be obtained to measure PTS relative to the mTA when considering slope‐reducing osteotomy.

**Level of Evidence:**

Level IV.

## Graphical abstract

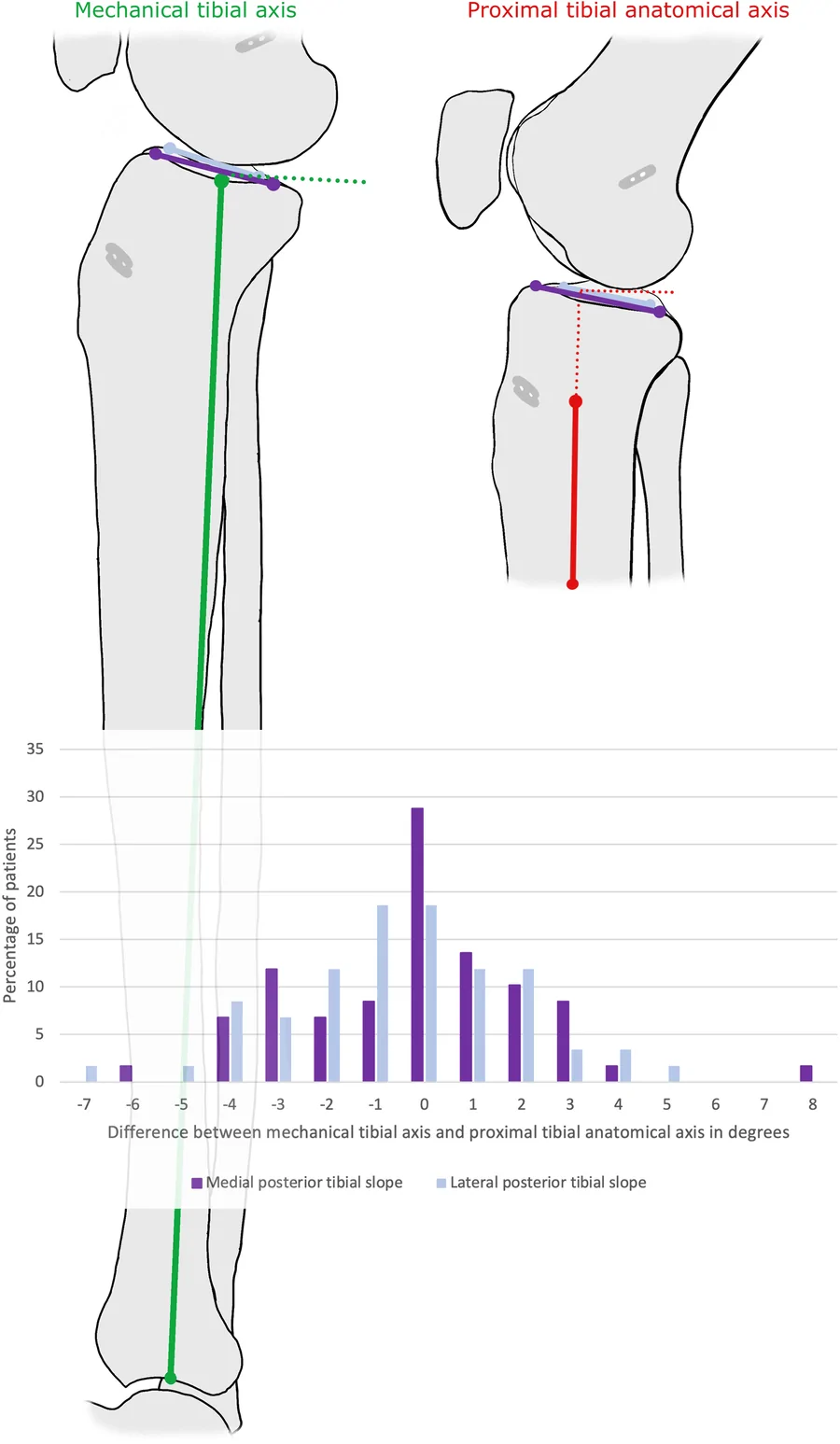

**Abbreviations**
ACL: anterior cruciate ligament
ATC: anterior tibial cortical axis
CI: confidence interval
ICC: intraclass correlation coefficient
IRB: institutional review board
LoA: limits of agreement
LTP: lateral tibial plateau
mTA: mechanical tibial axis
MTP: medial tibial plateau
NPV: negative predictive value
PCL: posterior cruciate ligament
PPV: positive predictive value
PTAA: proximal tibial anatomical axis
PTC: posterior tibial cortical axis
PTS: posterior tibial slope
SD: standard deviation

## INTRODUCTION

The posterior tibial slope (PTS) is a key determinant of knee biomechanics because it alters anterior‐posterior ligament loading. Even a modest 2° increase in PTS raises anterior cruciate ligament (ACL) strain and increases ACL reconstruction graft failure risk [8, 9, 30], whereas a decrease in PTS raises posterior cruciate ligament (PCL) strain, potentially compromising PCL reconstruction outcomes [2, 25]. Accurate PTS measurement is therefore essential when surgeons consider a slope‐changing osteotomy.

These effects are fundamentally mechanical in nature. Yet in clinical practice, PTS is usually measured relative to proximal anatomical axes rather than relative to the biomechanically relevant mechanical tibial axis (mTA) [10, 12, 18, 31]. This discrepancy is largely due to practical considerations, as proximal anatomical reference axes can be readily defined on standard lateral knee radiographs, whereas mechanical axis determination requires full‐length lateral lower‐leg radiographs [9].

Although multiple studies report moderate to strong correlations between proximal‐anatomical‐axis‐based and mechanical‐axis‐based PTS measurements [7, 12, 14, 22, 27], measurement errors up to 11° have been documented [7]. More importantly, it is still unclear whether these errors result in a systematic and unidirectional or bidirectional bias [7]. A systematic error, characterized by a consistent overestimation or underestimation of PTS depending on the axis used, could still allow for clinical applicability by establishing axis‐specific cut‐off values. In contrast, a bidirectional error introduces significant clinical risk; an overestimated PTS may result in an unnecessary slope‐reducing osteotomy, whereas an underestimated PTS may leave a clinically relevant deformity untreated.

Therefore, the purpose of this study was to determine whether proximal anatomical axes‐based PTS measurements can serve as reliable proxies for mTA‐based PTS measurements. The aim was to test the hypothesis that, despite strong linear correlation, proximal anatomical axes‐based PTS measurements display clinically relevant bidirectional measurement error (both under‐ and overestimation) when compared with mTA‐based PTS measurements, thereby limiting their clinical interchangeability.

## METHODS

### Study design

A retrospective multicenter observational study was conducted at three orthopaedic centres (OCON Centre for Orthopaedic Surgery and Sports Medicine, Hengelo, The Netherlands; Kliniek ViaSana, Mill, The Netherlands; and Martin‐Luther Hospital, Berlin, Germany). The study adhered to the Strengthening the Reporting of Observational Studies in Epidemiology guidelines [6].

### Patient selection

Patients with clinical failure after primary or revision ACL reconstruction were retrospectively identified from institutional electronic health records and screened for eligibility. Clinical failure was defined as ACL graft re‐rupture and/or pathological laxity (side‐to‐side instrumented Lachman ≥ 5 mm) and/or persistent instability [16]. Inclusion criteria were: age ≥ 18 years; a lateral knee radiograph with depiction of the proximal tibia of ≥15 cm below the knee joint [31]; a lateral full‐length lower‐leg radiograph capturing the entire tibia in a single exposure. Exclusion criteria were: malrotation with ≥5 mm tibial and/or femoral condyle offset or overlap of femoral condyles with the tibial plateau on either the lateral knee radiograph and/or lateral full‐length lower‐leg radiograph, in order to ensure the PTS is measured in equal sagittal planes between groups, and independent of the weight‐bearing state [13, 23, 32]; previous tibial osteotomies and/or fractures; advanced osteoarthritis (Kellgren–Lawrence ≥Grade 3) [19]. Consecutive cases were screened until the sample size determined by the a priori power analysis had been reached.

### Measurement technique

PTS measurements on the lateral knee and lower‐leg radiographs were performed digitally using Picture Archiving and Communication Systems.

Medial and lateral PTS were defined as the angle between the medial and lateral tibial plateau joint orientations, respectively, and a line perpendicular to a longitudinal tibial axis [26]; a positive value indicated an anterior–posterior downslope, and a negative value represented an anterior–posterior upslope.

The medial and lateral tibial joint orientations were defined by a line connecting the most proximal anterior and most proximal posterior point of the medial and lateral tibial plateau, respectively. Medial and lateral PTS angles were assessed relative to four longitudinal tibial axes (Figure 1): (1) the mTA; (2) the proximal tibial anatomical axis (PTAA); (3) the anterior tibial cortical axis (ATC); and (4) the posterior tibial cortical axis (PTC). The mTA was defined as the axis connecting the midpoints of the tibial plateau and tibial plafond on the full‐length lateral lower‐leg radiographs [26]. The PTAA was defined as the axis connecting mid‐cortical diameters of the tibia at points 5 and 15 cm distal from the knee joint line on the lateral knee radiographs [26, 36]. The ATC and PTC were defined as the axes connecting points on the anterior and posterior cortex, respectively, of the proximal tibial diaphysis at 5 and 15 cm distal to the knee joint line on the lateral knee radiographs [36].

**Figure 1:**
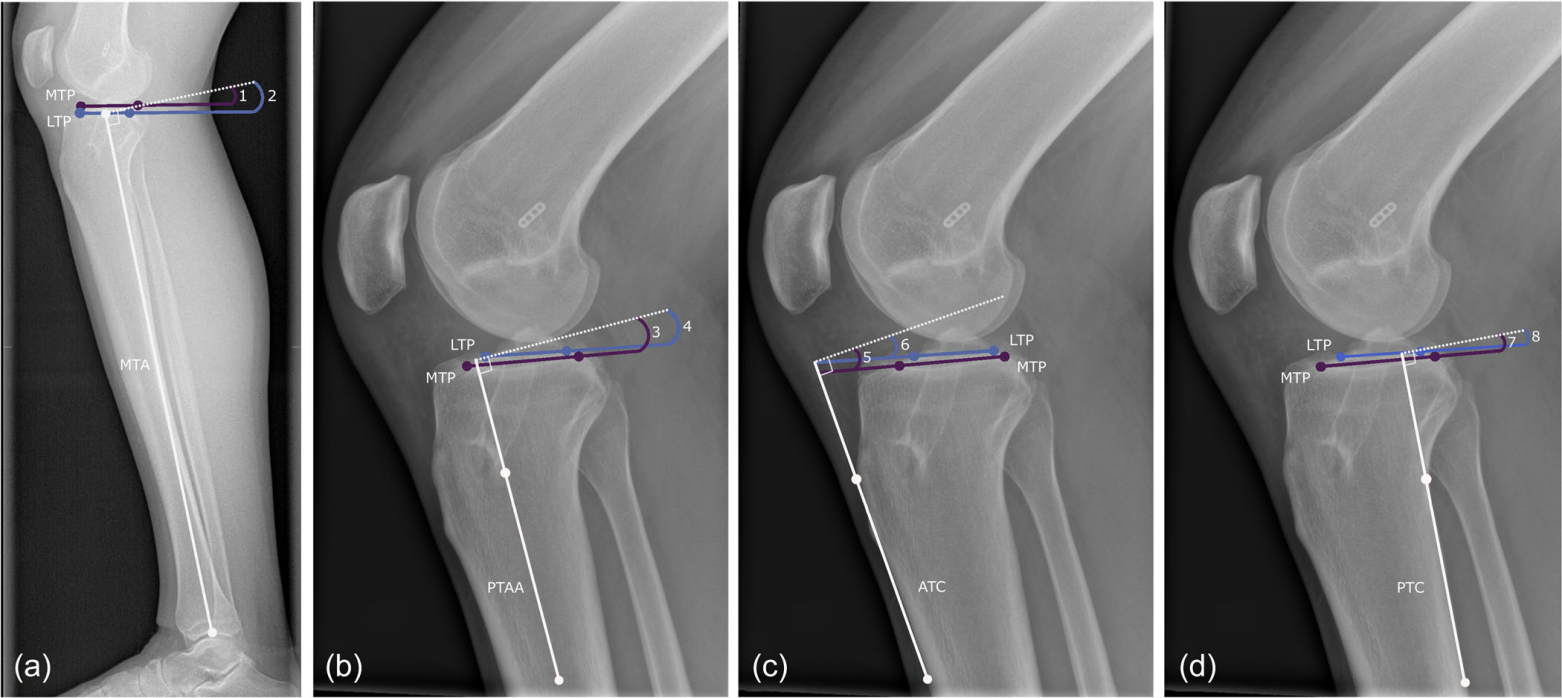
Posterior tibial slope (PTS) measurements on radiographs of a right knee. The medial and lateral PTS were defined by the angle between the line perpendicular to a longitudinal tibial axis (white dotted lines) and the medial tibial plateau (MTP; purple line) and lateral tibial plateau (LTP; blue line), respectively. (a) Mechanical tibial axis (mTA; white line) on a full‐length lateral lower‐leg radiograph, resulting in (1) the PTS between the MTP and mTA and (2) the PTS between the LTP and mTA. (b) Proximal anatomical tibial axis (PTAA; white line) on a lateral knee radiograph resulting in (3) the PTS between the MTP and PTAA and (4) the PTS between the LTP and PTAA. (c) Anterior tibial cortical axis (ATC; white line) on a lateral knee radiograph resulting in (5) the PTS between the MTP and ATC and (6) the PTS between the LTP and ATC. (d) Posterior tibial cortex axis (PTC; white line) on a lateral knee radiograph resulting in (7) the PTS between the MTP and PTC and (8) the PTS between the LTP and PTC.

### Statistical analysis

To assess the intra‐ and interobserver reliability of the PTS measurements, two experienced observers (one fellowship‐trained orthopaedic surgeon [B.C.B.] and one technical physician experienced in 2D and 3D leg alignment analysis and correction planning [Q.W.T.V]) independently performed radiographic measurements two times on 42 randomly selected cases, with a minimum 4‐week interval. Intra‐ and inter‐observer agreement for each PTS measurement was quantified with a two‐way random‐effects intraclass correlation coefficient (ICC) using absolute agreement and corresponding 95% confidence intervals (CIs). Reliability coefficients were classified as poor (<0.50), moderate (0.50–0.75), good (0.75–0.90) or excellent (>0.90) according to Koo and Li [20]. If observer reliability was deemed good‐to‐excellent, the first of the two readings from the primary observer (B.C.B.) was used in all subsequent analyses.

For each combination of plateau (medial and lateral) and axis (mTA, PTAA, ATC and PTC), the normality of continuous variables was assessed and confirmed using the Shapiro–Wilk test (*p* > 0.05). Paired comparisons between proximal anatomical axis‐based and mTA‐based PTS measurement were conducted using two‐tailed paired *t*‐tests and presented as mean ± standard deviation (SD) and range. Measurement agreement between proximal anatomical axis‐based and mTA‐based PTS measurements was characterized by Pearson's correlation coefficient (*r*) [28]. Since correlation alone does not imply measurement agreement, signed, absolute and mean measurement error were calculated between proximal anatomical axis‐based and mTA‐based PTS measurements and Bland–Altman analyses were performed (difference = mTA‐based PTS value − anatomical axis‐based PTS value). Signed measurement error was expressed as percentages and visualized using stacked bar charts representing 1° error bands. Absolute measurement error was reported as the percentage of patients with a ≥2° difference. Mean measurement error was presented as mean ± SD and range. For the Bland–Altman analyses, the mean bias (*Δ*), proportional bias and 95% limits of agreement (LoA = *Δ* ± 1.96·SD) were calculated for each comparison. Clinical interchangeability was defined a priori as LoA within 2°. Finally, the diagnostic accuracy of the conventional ≥12° PTAA‐based PTS cut‐off for slope‐reducing osteotomy with the mTA‐based PTS measurement as reference was determined, including sensitivity, specificity, positive predictive value (PPV) and negative predictive value (NPV).

A priori power analysis (G*Power 3.1.9.7) indicated that 59 paired measurements achieve 90% power (*β* = 0.10) to detect a 1° mean measurement error (SD = 2.32°) [7] with two‐tailed *α* = 0.05. All statistical tests were two‐sided, and *p* values < 0.05 were considered statistically significant. Statistical analyses were performed in SPSS software, version 30 (IBM Corp.).

## RESULTS

A total of 301 consecutive imaging sets were reviewed, of which 215 were excluded as the lateral knee radiograph depicted <15 cm of proximal tibia. A further 27 were excluded for malrotation (*n* = 12), prior tibial surgery (*n* = 7) or fracture (*n* = 8), leaving 59 cases for analysis.

### Observer reliability

Intra‐ and interrater reliability were considered good to excellent (≥0.933 for observer 1; ≥0.913 for observer 2 and ≥0.857 for interrater) for all radiographic measurement techniques (Table 1).

**Table 1:** Intra‐ and interrater reliability.

	Intrarater: observer 1	95% CI	Intrarater: observer 2	95% CI	Interrater	95% CI
Medial PTS‐mTA	0.965	0.935–0.981	0.985	0.944–0.984	0.983	0.968–0.991
Lateral PTS‐mTA	0.963	0.933–0.980	0.931	0.962–0.981	0.952	0.911–0.974
Medial PTS‐PTAA	0.937	0.886–0.965	0.913	0.844–0.952	0.938	0.884–0.966
Lateral PTS‐PTAA	0.933	0.879–0.963	0.922	0.860–0.958	0.864	0.711–0.932
Medial PTS‐ATC	0.960	0.926–0.978	0.935	0.882–0.965	0.941	0.888–0.969
Lateral PTS‐ATC	0.942	0.894–0.968	0.935	0.883–0.965	0.857	0.719–0.925
Medial PTS‐PTC	0.955	0.918–0.975	0.920	0.856–0.956	0.932	0.873–0.964
Lateral PTS‐PTC	0.959	0.925–0.978	0.923	0.862–0.958	0.904	0.803–0.951

Abbreviations: ATC, anterior tibial cortex; CI, confidence interval; mTA, mechanical tibial axis; PTC, posterior tibial cortex; PTAA, proximal tibial anatomical axis; PTS, posterior tibial slope.

### Measurement error in PTS using the PTAA versus the mTA

Medial and lateral PTS measured with the PTAA showed strong linear correlation with PTS measured with the mTA (medial *r* = 0.75; lateral: *r* = 0.76). There was no statistically significant difference in means for either plateau (medial 9.6 ± 3.4° vs. 9.4 ± 3.4°; lateral 10.3 ± 3.4° vs. 9.7 ± 3.4°; *p* = 0.675 and 0.106, respectively). A mean measurement error of 0.1 ± 2.4° (range: −5.7° to 7.6°) for the medial and −0.5 ± 2.4° (range: −6.6° to 5.2°) for the lateral plateau was observed (Table 2). Absolute measurement error showed ≥2° difference in 49.1% (medial) and 50.9% (lateral) of cases (Figure 2). The Bland–Altman analysis demonstrated a negligible mean bias but wide LoA between PTAA and mTA measurements (medial *Δ* = −0.13°, LoA = −4.89° to 4.62°; lateral *Δ* = −0.5°, LoA = −5.11° to 4.11°) (Figure A1). No proportional bias was detected (*p* = 0.816 [medial] and 0.857 [lateral]).

**Table 2:** Medial and lateral PTS values per axis.

Reference	Mean ± SD	Range	*p*	Mean measurement error ± SD	Range	Mean bias	95% LoA
Medial PTS							
mTA^a^	9.4 ± 3.4	0.4–17.3					
PTAA	9.6 ± 3.4	3.0–17.5	0.675	−0.1 ± 2.4	−5.7 to 7.6	−0.13	−4.89 to 4.62
ATC	12.8 ± 3.4	4.7–19.0	<0.001	−3.4 ± 2.6	−8.6 to 5.9	−3.4	−8.5 to 1.7
PTC	6.5 ± 3.5	−0.1 to 15.1	<0.001	3.0 ± 2.6	−2.9 to 10.4	2.96	−2.17 to 8.10
Lateral PTS							
mTA^a^	9.7 ± 3.4	1.8–18.5					
PTAA	9.6 ± 3.4	2.8–19.1	0.106	−0.5 ± 2.4	−6.6 to 5.2	−0.5	−5.11 to 4.11
ATC	13.5 ± 3.5	6.2–21.1	<0.001	−3.7 ± 2.6	−9.9 to 2.7	−3.71	−8.77 to 1.35
PTC	7.1 ± 3.5	−0.4 to 17.0	<0.001	2.7 ± 2.4	−3.6 to 7.8	2.66	−2.01 to 7.32

*Note*: All values in degrees (°).

Abbreviations: ATC, anterior tibial cortex; LoA, limits of agreement; mTA, mechanical tibial axis; PTAA, proximal tibial anatomical axis; PTC, posterior tibial cortex; PTS, posterior tibial slope; SD, standard deviation.

a mTA was used as reference value.

**Figure 2:**
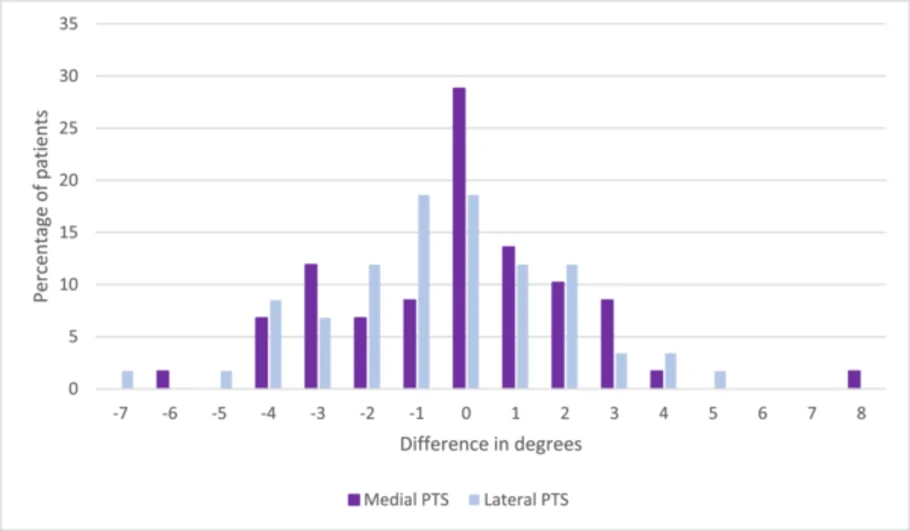
Frequency distribution of paired signed measurement error between PTAA and mTA‐based PTS measurements. Columns represent the percentage of knees falling within each 1° bin; negative values indicate that PTAA produced a larger slope than mTA. mTA, mechanical tibial axis; PTAA, proximal tibial anatomical axis; PTS, posterior tibial slope.

### Measurement error in PTS using the ATC versus the mTA

Medial and lateral PTS measured with the ATC produced significantly higher mean PTS values compared to the PTS measured with the mTA (medial: 12.8 ± 3.4° vs. 9.4 ± 3.4°; lateral: 13.5 ± 3.5° vs. 9.7 ± 3.4°; *p* < 0.001). Despite strong linear correlation (medial: *r* = 0.72; lateral: *r* = 0.72), a mean measurement error of −3.4 ± 2.6° (range: −8.6° to 5.9°) for the medial and −3.7 ± 2.6° (range: −9.9° to 2.7°) for the lateral plateau was observed (Table 2). Absolute measurement error showed ≥2° difference in 83% (medial) and 86% (lateral) of cases (Figure 3). The Bland–Altman analysis demonstrated wide LoA between ATC and mTA measurements (medial *Δ* = −3.4°, LoA = −8.5° to 1.7°; lateral *Δ* = −3.71°, LoA = −8.77° to 1.35°) (Figure S2). No proportional bias was detected (*p* = 0.972 [medial] and 0.849 [lateral]).

**Figure 3:**
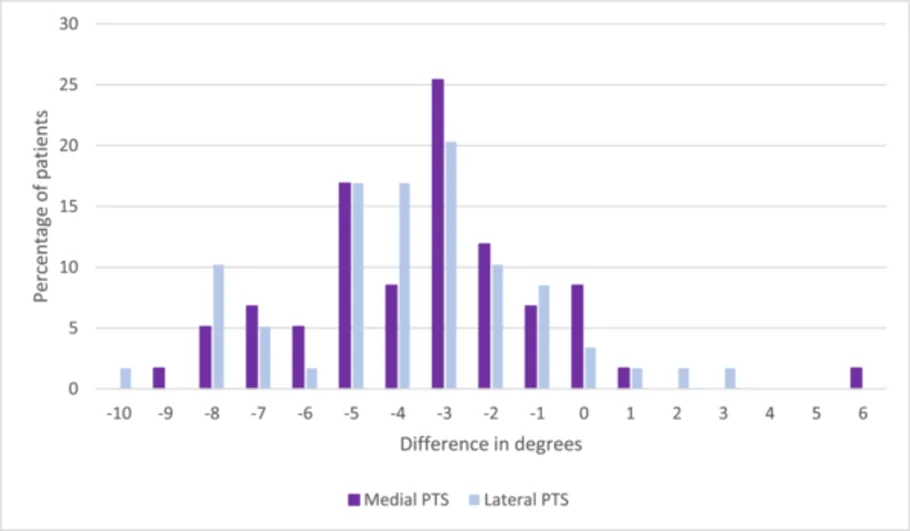
Frequency distribution of paired signed measurement error between ATC and mTA‐based PTS measurements. Columns represent the percentage of knees falling within each 1° bin; negative values indicate that ATC produced a larger slope than mTA. ATC, anterior tibial cortical axis; mTA, mechanical tibial axis; PTS, posterior tibial slope.

### Measurement error in PTS using the PTC versus the mTA

Medial and lateral PTS measured with the PTC produced significantly lower mean PTS values compared to the PTS measured with the mTA (medial: 6.5 ± 3.5° vs. 9.4 ± 3.4°; lateral: 7.1 ± 3.5° vs. 9.7 ± 3.4°; *p* < 0.001). Linear correlations remained strong (medial: *r* = 0.71; lateral: *r* = 0.76), but a mean measurement error of 3.0 ± 2.6° (range: −2.9° to 10.4°) for the medial and 2.7 ± 2.4° (range: −3.6° to 7.8°) for the lateral plateau was observed (Table 2). Absolute measurement error showed ≥2° difference in 82% (medial) and 78% (lateral) of cases (Figure 4). The Bland–Altman analysis demonstrated a wide LoA between PTC and mTA measurements (medial *Δ* = 2.96°, LoA = −2.17° to 8.10°; lateral *Δ* = 2.66, LoA = −2.01° to 7.32°) (Figure S3). No proportional bias was detected (*p* =  0.972 [medial] and 0.845 [lateral]).

**Figure 4:**
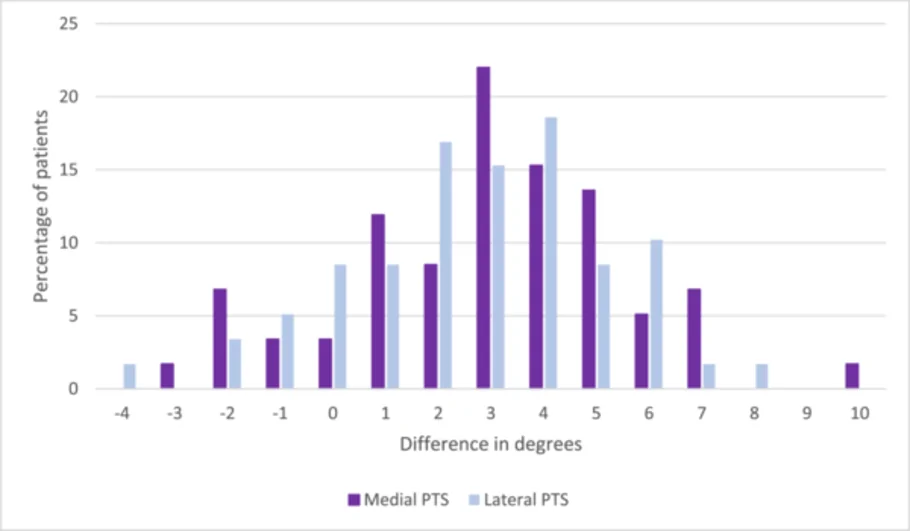
Frequency distribution of paired signed measurement error between PTC and mTA‐based PTS measurements. Columns represent the percentage of knees falling within each 1° bin; negative values indicate that PTC produced a larger slope than mTA. mTA, mechanical tibial axis; PTC, posterior tibial cortical axis; PTS, posterior tibial slope.

### Diagnostic accuracy with cut‐off threshold of ≥12° PTAA‐based PTS

Using the conventional threshold of ≥12° PTS based on the PTAA to decide if a slope reducing high tibial osteotomy is indicated, 8.5% of medial, 18.2% of lateral PTS measurements and 19.5% of either one or both plateau's were classified as false positive, whereas 10.4%, 10.0% and 13.2%, respectively, were false negative when compared to PTS based on the mTA [35]. The NPV was 72% (95% CI = 0.491–0.875) and the PPV 80.5% (95% CI = 0.660–0.898) for elevated medial and/or lateral tibial PTS (Table 3).

**Table 3:** Diagnostic accuracy of the conventional ≥12° PTAA‐based PTS cut‐off for slope‐reducing osteotomy with mTA‐based PTS measurement as reference.

	Medial PTS	95% CI	Lateral PTS	95% CI	Medial and/or lateral PTS	95% CI
Accuracy	0.847	0.735–0.918	0.797	0.677–0.880	0.780	0.659–0.867
Sensitivity	0.636	0.354–0.848	0.579	0.363–0.769	0.619	0.409–0.792
Specificity	0.896	0.778–0.955	0.900	0.769–0.960	0.868	0.727–0.942
PPV	0.583	0.319–0.807	0.733	0.480–0.891	0.722	0.491–0.875
NPV	0.915	0.801–0.967	0.818	0.680–0.904	0.805	0.660–0.898

Abbreviations: CI, confidence interval; mTA, mechanical tibial axis; NPV, negative predictive value; PPV, positive predictive value; PTAA, proximal tibial anatomical axis; PTS, posterior tibial slope.

## DISCUSSION

The most important findings of the present study are that, despite strong linear correlations, PTS measurements based on proximal anatomical axes exhibit substantial bidirectional measurement error (both under‐ and overestimation) outside clinically acceptable LoA when compared with mTA‐based PTS measurements. An absolute measurement error ≥2° was observed in approximately half of the PTAA readings relative to the mTA readings. Using the conventional ≥12° cut‐off on PTAA‐based PTS values would have misclassified approximately one in three knees (19.5% false‐positive and 13.2% false‐negative) relative to the mTA‐based PTS reference values. Because of the limited diagnostic accuracy, highlighted by the wide LoA of approximately 5° substantially exceeding the clinically relevant threshold of 2°, a high PTAA‐based PTS should not be used in isolation; confirmation with mTA‐based PTS measurements on a lateral lower‐leg radiograph is warranted. These data call into question the routine reliance on anatomical‐axis‐based PTS measurement from standard lateral knee radiographs and support mTA‐based PTS measurement on lateral lower‐leg radiographs whenever PTS magnitude informs treatment decisions.

The present study confirms previous findings indicating that lateral knee radiographs provide imprecise estimates of PTS [1, 12, 18, 22]. Dean et al. likewise reported ≥2° errors in half of their patient population, as well as a strong correlation and a non‐significant difference in means of <1° [7]. However, as Dean et al. considered only the absolute difference in means, it remains unclear wether this reflects a systematic unilateral bias or a non‐systematic bilateral bias where both over‐ and underestimation of PTS was present. Nonetheless, Dean et al. concluded that PTAA‐based PTS measurement could act as a proxy for mTA‐based PTS measurement. This, however, might be based on the misconception that strong correlation equals clinical interchangeability. The current study provides novel insight by demonstrating that, despite strong correlations and non‐significant difference in means, there is a bidirectional error between the different measurement methods with a much wider measurement variability than clinically acceptable. Given that even a 2° increase in PTS has been shown to significantly elevate strain on the native or reconstructed ACL graft, such measurement error is of clear clinical relevance [2]. The current study highlights the risk of unnecessary slope‐reducing osteotomies in case of overestimation and the potential for untreated clinically relevant deformities in case of underestimation of PTS measurements based on proximal anatomical axes.

A recent international Delphi consensus study acknowledged these limitations of PTAA‐based PTS measurements and the lack of a universally applicable cut‐off value—most of which are derived from studies using proximal anatomical axes [33]. Our findings provide a potential explanation: the bidirectional, patient‐specific error outside clinically acceptable LoA limits the reliability of PTAA‐based measurements. While PTAA‐based PTS measurements remain common due to their feasibility on standard lateral knee radiographs, we argue that they should not be used in the work‐up for slope‐reducing osteotomy. We therefore advocate for the routine use of single‐exposure, full‐length lateral tibial radiographs allowing mTA‐based measurement, both clinically and in future studies aiming to establish valid cut‐off values. Given the clinically relevant absolute differences between anatomical‐ and mechanical‐axis‐based PTS measurements, anatomical‐axis‐based cut‐off values from previous literature may not apply to mTA‐based measurements, and therefore new reference values must be defined.

The bidirectional nature of the measurement error likely reflects individual variation in tibial anatomy. The tibial tuberosity geometry, around which the PTAA is defined, varies considerably between individuals, altering PTAA orientation [29], whereas anterior tibial bowing changes the course of the cortical (and central proximal anatomical) axes [14, 15]. Because these variations differ in magnitude and direction between patients, the resulting error can be positive or negative and cannot be corrected by a systematic constant offset. Furthermore, the absence of proportional bias indicates that these errors do not consistently increase or decrease with the size of the measurement, making them inherently unpredictable, corroborating our finding that one‑third of cases were misclassified. Accurate assessment of PTS should therefore be based on the mTA using full‐length tibial radiographs.

A slope‐reducing high tibial osteotomy is increasingly proposed for ACL reconstruction failure when the PTAA‐based measurement PTS is ≥12°. This PTAA‐based PTS is defined by midpoints at 5 and 15 cm distal to the joint line [35]. However, in our multicenter cohort, only 29% of lateral knee radiographs included at least 15 cm of proximal tibia, limiting the applicability of this method in routine clinical practice. Moreover, up to 21.5% of patients would have been undertreated, and 35.2% overtreated. Therefore, it might be better to obtain full‐length single‐exposure lateral lower‐leg radiographs whenever precise PTS measurement relative to the mechanical axis is needed.

Recent systematic reviews and meta‐analyses investigating the relationship between increased PTS and ACL injury risk pool PTS values based on the PTAA, ATC and PTC—sometimes even without distinction between radiograph and MRI measurements— without stratification [5, 8, 11, 17, 21, 24, 30, 34, 37, 38]. Given the ~6° spread between anatomical axes observed in the present and other previous studies [4, 31, 36], such aggregation increased heterogeneity and may dilute true effect estimates. Future reviews should analyze each axis and imaging modality separately, or, preferably, include only mTA‐based PTS measurements.

Several limitations should be considered. First, despite the use of standardized radiographic protocols, subtle differences in patient positioning may have influenced the accuracy of PTS measurements [3, 27]. To mitigate this, radiographs with evident projectional distortion were excluded to minimize potential measurement bias. Second, the study cohort primarily consisted of patients with failed ACL reconstructions, potentially restricting generalizability to other patient groups. However, the multicenter design, with three independent institutions, enhances external validity and reduces centre‐specific treatment bias. Third, the retrospective design with enrolment stopped once the a‐priori sample size of 59 radiographs was reached, which may introduce selection bias. This risk is partially offset by the demonstrated measurement reliability: duplicate assessments by two blinded, experienced observers yielded excellent ICC (≥0.93), supporting the internal validity of the data.

## CONCLUSION

Despite strong correlation, PTS measured relative to proximal anatomical axes on routine lateral knee radiographs shows substantial, bidirectional measurement error outside clinically acceptable LoA compared to PTS measured relative to the mTA on full‐length lateral lower‐leg radiographs. Proximal anatomical axes‐based PTS measurements are therefore not interchangeable with mechanically relevant mTA‐based PTS measurements. A single‐exposure, full‐length lateral lower‐leg radiograph should be obtained so that PTS can be measured relative to the mTA when considering slope‐reducing osteotomy.

## AUTHOR CONTRIBUTIONS

All the authors have been actively involved in the planning and enactment of the study, and are involved in the writing of the submitted paper. The first and second authors (Barbara C. Boer and Quinten W. T. Veerman) also performed the radiological measurements.

## CONFLICT OF INTEREST STATEMENT

The authors declare no conflicts of interest.

## ETHICS STATEMENT

The Institutional Review Board (IRB) of the Centre for Orthopaedic Surgery and Sports Medicine OCON approved this study (IRB nr: OCON2024002).

## Figures and Tables

**Figure A1:**
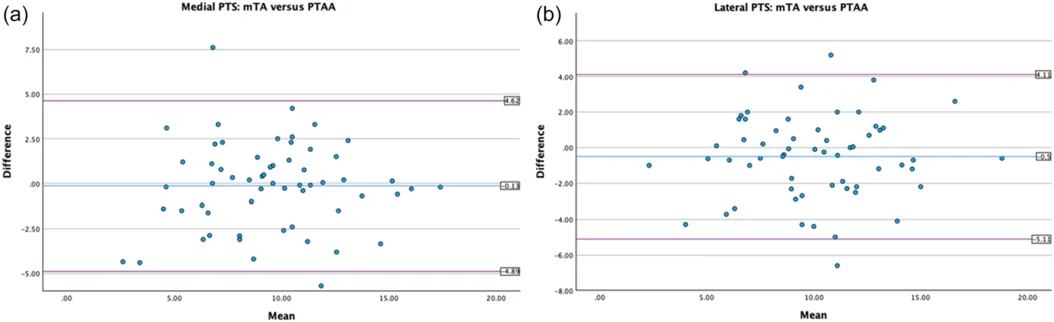
Bland–Altman analysis: agreement of PTS measurements between the proximal tibial anatomical axis (PTAA) and the mechanical tibial axis (mTA). (a) medial PTS; (b) lateral PTS. Blue line: mean bias (*Δ*). Purple lines: upper and lower 95% limits of agreement (LoA = *Δ* ± 1.96·SD). PTS, posterior tibial slope.

**Figure A2:**
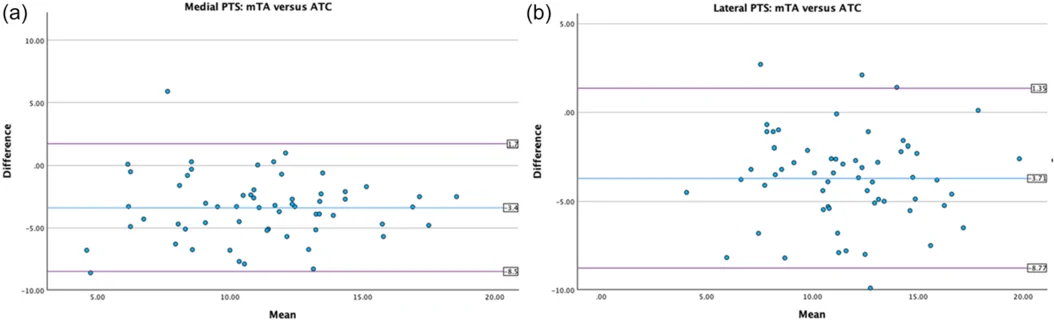
Bland–Altman analysis: agreement of PTS measurements between the anterior tibial cortex (ATC) and the mechanical tibial axis (mTA). (a) medial PTS; (b) lateral PTS. Blue line: mean bias (*Δ*). Purple lines: upper and lower 95% limits of agreement (LoA = *Δ* ± 1.96·SD). PTS, posterior tibial slope.

**Figure A3:**
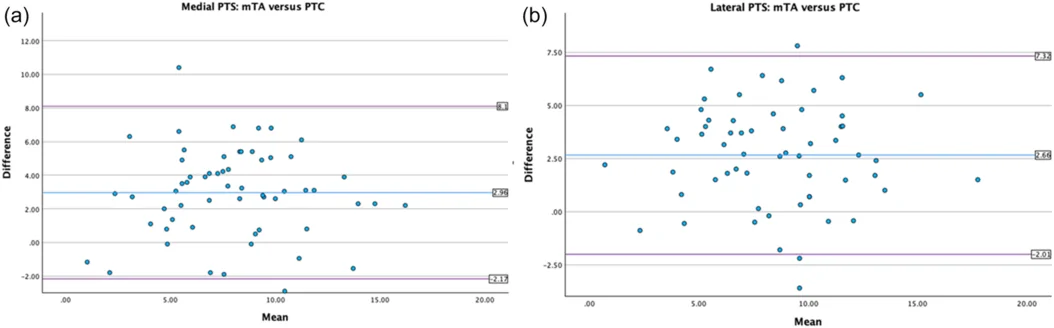
Bland–Altman analysis: agreement of PTS measurements between the posterior tibial cortex (PTC) and the mechanical tibial axis (mTA). (a) medial PTS; (b) lateral PTS. Blue line: mean bias (*Δ*). Purple lines: upper and lower 95% limits of agreement (LoA = *Δ* ± 1.96·SD). PTS, posterior tibial slope.

## Data Availability

The data that support the findings of this study are available from the corresponding author upon reasonable request.
